# Betaine Supplementation Enhances Lipid Metabolism and Improves Insulin Resistance in Mice Fed a High-Fat Diet

**DOI:** 10.3390/nu10020131

**Published:** 2018-01-26

**Authors:** Jingjing Du, Linyuan Shen, Zhendong Tan, Peiwen Zhang, Xue Zhao, Yan Xu, Mailing Gan, Qiong Yang, Jideng Ma, An’an Jiang, Guoqing Tang, Yanzhi Jiang, Long Jin, Mingzhou Li, Lin Bai, Xuewei Li, Jinyong Wang, Shunhua Zhang, Li Zhu

**Affiliations:** 1 College of Animal Science and Technology, Sichuan Agricultural University, Chengdu 625014, China; 18111521611@163.com (J.D.); 15181216032@163.com (L.S.); tzdabc123456@163.com (Z.T.); sicau_zhangpeiwen@163.com (P.Z.); 18227588896@163.com (X.Z.); yiyazhi@yahoo.com (Y.X.); 18299095425@139.com (M.G.); jideng_ma@sina.com (J.M.); 13540384633@139.com (A.J.); tyq003@163.com (G.T.); longjin@sicau.edu.cn (L.J.); mingzhou.li@sicau.edu.cn (M.L.); blin16@126.com (L.B.); xuewei.li@sicau.edu.cn (X.L.); 2 Department of Animal Husbandry and Veterinary Medicine, Chengdu Agricultural College, Chengdu 611100, China; 18180008728@163.com; 3 College of Life and Biology Science, Sichuan Agricultural University, Chengdu 611130, China; jiangyz04@163.com; 4 Chongqing Academy of Animal Science, Chongqing 402460, China; kingyou@vip.sina.com

**Keywords:** betaine, obesity, WAT, insulin resistance, intramyocellular lipids

## Abstract

Obesity is a major driver of metabolic diseases such as nonalcoholic fatty liver disease, certain cancers, and insulin resistance. However, there are no effective drugs to treat obesity. Betaine is a nontoxic, chemically stable and naturally occurring molecule. This study shows that dietary betaine supplementation significantly inhibits the white fat production in a high-fat diet (HFD)-induced obese mice. This might be due to betaine preventing the formation of new white fat (WAT), and guiding the original WAT to burn through stimulated mitochondrial biogenesis and promoting browning of WAT. Furthermore, dietary betaine supplementation decreases intramyocellular lipid accumulation in HFD-induced obese mice. Further analysis shows that betaine supplementation reduced intramyocellular lipid accumulation might be associated with increasing polyunsaturated fatty acids (PUFA), fatty acid oxidation, and the inhibition of fatty acid synthesis in muscle. Notably, by performing insulin-tolerance tests (ITTs) and glucose-tolerance tests (GTTs), dietary betaine supplementation could be observed for improvement of obesity and non-obesity induced insulin resistance. Together, these findings could suggest that inhibiting WAT production, intramyocellular lipid accumulation and inflammation, betaine supplementation limits HFD-induced obesity and improves insulin resistance.

## 1. Introduction

White adipose tissue (WAT) is mainly located in the subcutaneous fat (SAT) and visceral fat, which is an important secretory and energy metabolizing organ [1]. However, a large body of work indicates that SAT is the largest and least harmful adipose depot to store excess lipids. Accumulation of visceral fat significantly increases the risk of metabolic disease and mortality across ethnicities, even in individuals with a normal body mass index [2,3,4,5]. Notably, survival of several cancers depends on fatty acid oxidation [6,7]. Particularly, adipocytes can secrete a large number of disease-promoting exosomes that contain special microRNAs, proteins and mRNAs to establish a distant pro-metastatic niche [8,9,10]. Ikrame et al. [11] reported that mature adipocyte-derived exosomes promote melanoma aggressiveness through fatty acid oxidation. Deng et al. [12] suggested that adipose tissue exosome-like vesicles mediate activation of macrophage-induced insulin resistance. Recently, Cheng et al. [13] found that perivascular adipose tissue-conditioned medium induced adventitial fibroblast migration, which may be associated with the pathogenesis of neointimal formation.

Excess energy due to a calorie-dense diet or disturbance of lipid metabolism, in humans and animals, is normally stored as fat in WAT that contains very few mitochondria [1,14,15,16]. Therefore, accumulation of WAT quickly leads to weight gain and even obesity. Nowadays, obesity represents a fast-growing global health problem, currently affecting 90 million people in China and more than 30% of the western population [11,17,18]. Studies show that obesity significantly increases the risk of several pathological conditions, including nonalcoholic fatty liver disease, atherosclerosis, hypertension, chronic inflammation, type 2 diabetes and certain types of cancers [19,20,21,22,23]. During the past decade, great strides have been made in understanding the development of WAT. Many obesity treatments like physical exercise and reduction of energy-dense food intake are also being encouraged to regulate the balance between caloric intake and energy expenditure. Nevertheless, the molecular mechanisms of adipose development remain unclear. More importantly, so far, no effective drugs are available to treat obesity.

Betaine, a naturally occurring molecule found naturallyin plants, microorganisms and animals, is a component of many common foods such as shellfish, wheat, beets and spinach. Additionally, betaine is an amino acid (trimethyl-glycine) present in most organisms and is an obligatory intermediate in the catabolism of choline [24]. The typical daily intake of betaine ranges from 1 to 2.5 g/day in humans [25]. Orally-administered betaine is retained systemically and can circulate in a steady state [26]. Recently, many studies have suggested that betaine supplementation improves power performance, attenuates Alzheimer-like pathological changes and memory deficits induced by homocysteine, and protects against renal injury induced by cadmium intoxication [27,28,29]. More recently, Jenna et al. [30] found that betaine supplementation enhances anabolic endocrine and Akt signaling in response to acute bouts of exercise. Wang et al. [31] reported that betaine attenuates hepatic steatosis by reducing methylation of the microsomal triglyceride transfer protein (MTTP) promoter and elevating genomic methylation in mice on a high-fat diet. Particularly, Asma et al. [32] proved that dietary betaine supplementation improves glucose homeostasis and reduces hepatic lipid accumulation. These results led to the hypothesis that betaine could be used as a therapeutic agent for treatment for obesity and obesity-induced complications. Exploration of the effect of betaine on white fat formation in vitro and in vivo, intramyocellular lipid accumulation and insulin resistance, and the possibility of the use of betaine in improving lipid metabolism disorders and glucose homeostasis, particularly in the treatment of obesity-induced clinical symptoms such as hyperglycemia, inflammation and non-alcoholic fatty liver disease follows.

## 2. Materials and Methods

### 2.1. Animal Treatments

All animal studies were carried out in accordance with the U.K. Animals (Scientific Procedures) Act, 1986 and associated guidelines. 40 Kunming mice (female, six weeks old, 19–20 g) were treated with a high-fat diet (HFD: 40.4% fat, 14.6% protein, 45.2% carbohydrate by energy) or normal chow (NCW: 13.2% fat, 23.2% protein, 63.6% carbohydrate by energy) for 13 weeks, respectively. Additionally, mice were housed at 22–24 °C and given free access to water, under controlled conditions of light and temperature. To evaluate the effect of betaine on HFD-induced obesity and hyperglycemia, 12 HFD- and 12 NCW-fed mice were treated with or without 1% (weight/volume, *W*/*V*) betaine (Sigma, St. Louis, MO, USA) in water according to a previous report [32]. Moreover, diabetes induced in female Kunming mice fed a HFD for 30 days was treated by intraperitoneal injection with 200 mg/kg streptozotocin [33] (STZ; Sigma-Aldrich, St. Louis, MO, USA). Blood glucose levels were measured using an Accu Check Advantage Glucometer (Roche, Dublin, Ireland, cat.06583261001).

### 2.2. Glucose and Insulin Tolerance Tests

To finish the glucose-tolerance test (GTT), 6 overnight-fasted mice were given intraperitoneal injections of 2 mg glucose/g body weight, as previously described [34]. 6 mice fasted for 4 h and then were treated by 0.5 mU insulin/g body weight by intraperitoneal injection, for insulin-tolerance tests (ITT). Blood was obtained from the tail vein before the injection and at 0, 15, 30, 60, and 90 min after the injection. Blood glucose was measured using an Accu Check Advantage Glucometer.

### 2.3. Serum-Sample Analysis

Briefly, to collect serum, blood samples were separated by centrifugation at 3000× *g* for 20 min at 4 °C. Serum samples were kept at −20 °C until further analysis. Then, serum levels of alanine transaminase (ALT), aspartate aminotransferase (AST), triglycerides (TG), cholesterol (TC) and low-density lipoprotein (LDL), high-density lipoprotein (HDL) and free-fatty acids (FFA) were determined by using commercial kits according to the manufacturers’ instruction.

### 2.4. Determination of Intramuscular Fat (IFM) and Fatty Acid Composition

Briefly, the same muscle samples from mice were collected immediately and stored at −20 °C. IMF then was determined as the percentage of fat extracted from 2 g of fresh tissue by the Soxhlet petroleum-ether extraction method [35]. Fatty acids were separated and determined according to previously published protocols [36,37]. The analysis was performed in triplicate for each sample.

### 2.5. Cell Culture

A growth medium containing Dulbecco’s modified Eagle’s medium (DMEM, Gibco, Carlsbad, CA, USA) with 10% fetal bovine serum held 3T3-L1 cells (Stem Cell Bank, Chinese Academy of Science) maintained at 37 °C, 5% CO_2_ before being induced to differentiate. To induce differentiation, the medium was switched to a differentiation medium containing 10% FBS, 0.5 mM 3-isobutyl-1-methylxanthine, 1 μM dexamethasone, and 5 μg/mL insulin. The medium was replaced every other day with DMEM containing 10% FBS and 5 μg/mL insulin, and the process was maintained until day 6. Additionally, to explore the effect of betaine on adipocyte proliferation and differentiation, cells were treated with or without 20 mM betaine.

### 2.6. Cell Proliferation Assay by CCK-8 and EdU Proliferation Analysis

Briefly, cells seeded in 96-well plates were treated with or without 20 mM betaine, and then Cells proliferation (containing control cells) at 0 h, 24 h, 48 h and 72 h were assessed by a Cell Counting kit 8 (CCK-8, Beyotime, Shanghai, China). Briefly, 36 h post-treatment, for EdU proliferation analysis, 3T3-L1 cells were treated with 15 μM ethynyldeoxyuridine (EdU) (RiboBio, Guangzhou, China) and incubated for a further 24 h. Edu staining was done according to the manufacturer’ protocol. Images were captured using an OLYMPUS IX53 microscope (OLYMPUS, Tokyo, Japan).

### 2.7. Oil Red-O Staining and Triglyceride Assay

The 6th day of differentiation had 3T3-L1 cells treated with 20 mM betaine washed three times with PBS, and fixed in 10% formalin for 30 min. The fixed samples were stained with 0.5% Oil Red O for 1.5 h at room temperature. Furthermore, muscle tissue preparations were performed as previously described and then stained with Oil Red O [33]. Images were captured using an OLYMPUS IX53 microscope (OLYMPUS, Tokyo, Japan). Stained cells were eluted with isopropanol for 20 min, for a 3T3-L1 cells triglyceride assay, and the optical density (OD) values were detected with a spectrophotometer at a wavelength of 510 nm.

### 2.8. Quantitative PCR

Briefly, as previously reported [38], total cellular RNA was extracted using TRIzol Reagent (Invitrogen, Carlsbad, CA, USA) according to the manufacturer’s instructions. Reverse transcription of mRNA was performed using a commercial kit (TaKaRa, Dalian, China), following the manufacturer’s protocol. Quantitative PCR was performed using the SYBR Premix Ex Taq kit (TaKaRa, Dalian, China) on a CFX96 system (Bio-Rad, Richmond, CA, USA). Relative expression levels of mRNAs were calculated using the 2^−ΔΔ*C*t^ method. The primer sequences used for quantitative real-time polymerase chain reaction (qRT-PCR) are listed in the Supplementary Table S1.

### 2.9. Sttistical Analysis

Each experiment was carried out in triplicate and all quantitative results are represented as means ± standard error (SE). SPSS 22.0 software (Chicago, IL, USA) was used for statistical analysis. Comparisons between the two parametric groups were made using student’s *t*-test. A value of *p* < 0.05 indicated a significant difference.

## 3. Results and Discussion

### 3.1. HFD Feeding Induced Obesity and Altered Metabolic Syndrome

**Figure 1:**
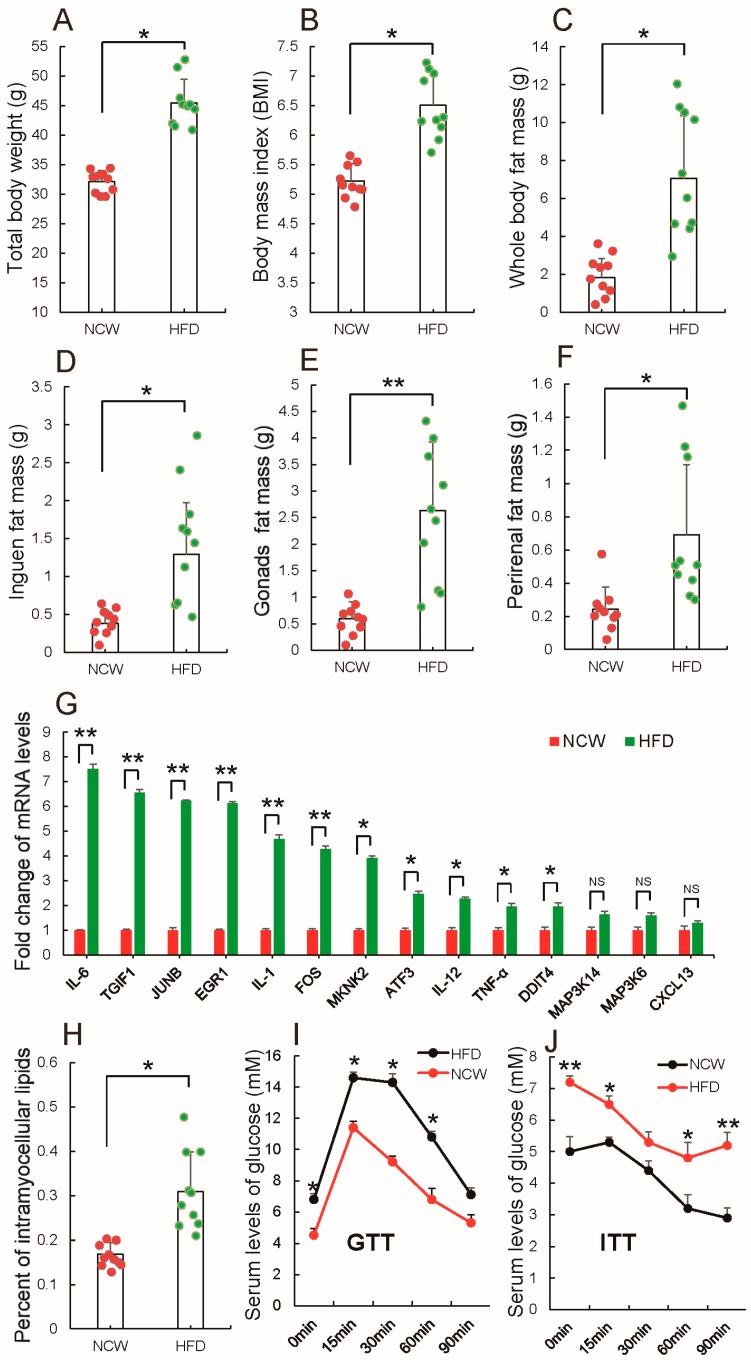
High-fat diet (HFD) feeding induces obesity. Following 13 weeks of normal chow (NCW)- or HFD-feeding in Kunming mice, (**A**) body weight; (**B**) body mass index (BMI); (**C**) whole body fat mass; (**D**) inguen fat mass; (**E**) gonads fat mass; (**F**) perirenal fat mass; and (**G**) the mRNA levels of inflammatory stress-related genes in adipose tissues were measured. Moreover, (**H**) the authors quantified intramyocellular lipids located in the same leg muscle tissues; performed (**I**) glucose-tolerance test (GTT) and (**J**) insulin-tolerance test (ITT). All results are presented as means ± standard error (SE). *n* = 10. * *p* < 0.05; ** *p* < 0.01; NS, no significance.

Previous studies suggested that consumption of a high-fat diet (HFD) rapidly reprograms systemic metabolism and, particularly, causes obesity [38,39]. Here, the effect of HFD on mice, after Kunming mice were fed by HFD or normal chow (NCW) for 13 weeks was evaluated. Figure 1A–C shows body weight, body mass index (BMI) and whole body fat mass were significantly higher in HFD-fed mice than NCW-fed mice. Further analysis showed that HFD-fed mice gained more inguinal fat (Figure 1D), gonadal fat (Figure 1E) and perirenal fat (Figure 1F), all of which were significantly increased when compared to NCW-fed mice, as reported also by Jeffery et al. [40]. Furthermore, consistent with previous findings that the expansion of adipose tissues in overweight or obese humans induced inflammation [41,42,43], qRT-PCR analysis showed that genes involved in promoting inflammation were expressed at high levels in adipose tissues of HFD-fed mice (Figure 1G). All results indicate that HFD feeding induced obesity.

Interestingly, it was observed that HFD feeding significantly increased accumulation of intramyocellular lipids as compared to NCW feeding (Figure 1H). Recently, it was demonstrated that the level of intramyocellular lipids can be used as a marker of insulin resistance, especially in type 2 diabetes mellitus [44]. Therefore, glucose levels and insulin sensitivity in HFD- and NCW-fed mice were evaluated subsequently. Figure 1I shows the higher levels of fasting blood glucose (at 0 min) were observed in HFD-fed mice. Glucose-tolerance tests (GTTs) show that glucose levels increased more in HFD-fed mice than NCW-fed mice (Figure 1I). Similarly, higher glucose levels at 0, 15, 30, 60, and 90 min of insulin-tolerance tests (ITTs) were found in HFD-fed mice, indicating that a HFD impaired glucose intolerance and decreased insulin sensitivity (Figure 1J). Taken together, these results demonstrate that a HFD feeding could induce obesity and alter metabolic syndrome, such as increasing white fat (WAT) production, lipid accumulation in muscle, and insulin resistance.

### 3.2. Betaine Supplementation Inhibited White Fat Production in HFD-Induced Obese Mice

**Figure 2:**
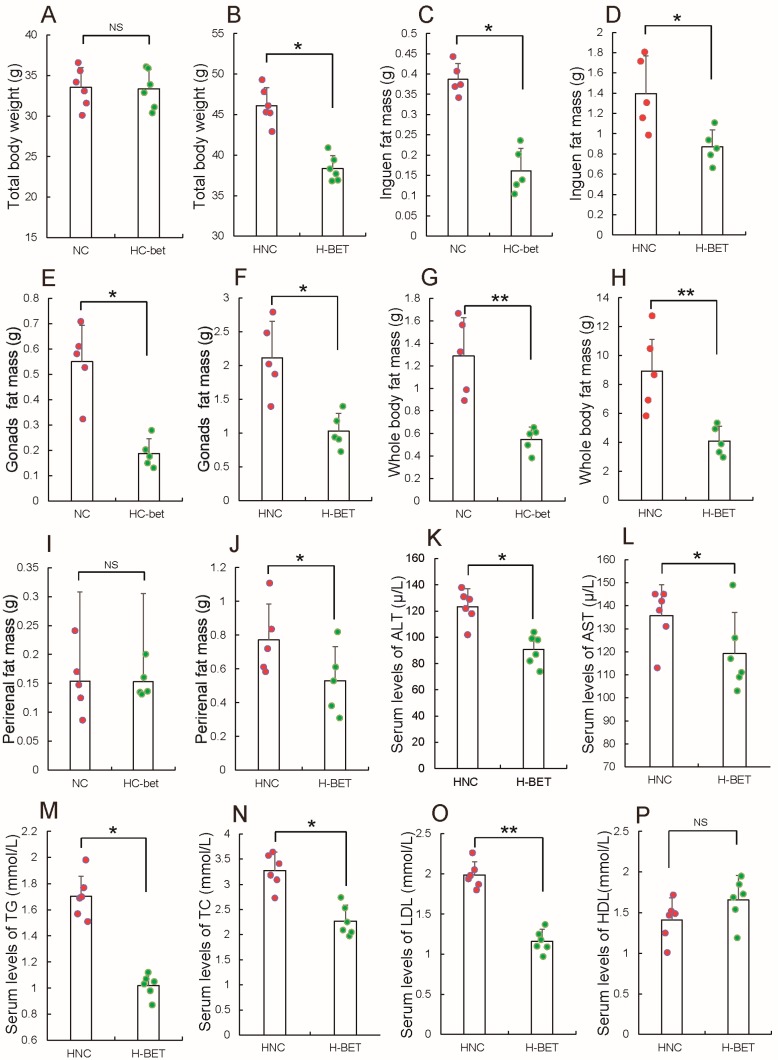
Betaine supplementation limits high-fat diet (HFD)-induced obesity. Following mice fed with HFD or NCW, treated with or without 1% betaine in water, (**A**,**B**) body weight in normal chow (NCW)-fed mice treated without (NC) or with (HC-bet) betaine, HFD feeding mice treated without (HNC) or with (H-BET) betaine were measured. Then, (**C**,**D**) inguen fat mass; (**E**,**F**) gonads fat mass; (**G**,**H**) whole body fat mass and (**I**,**J**) perirenal fat mass in NC or BET were measured. Additionally, after HFD-fed and NCW-fed mice were treated with or without 1% betaine in water, serum levels of (**K**) alanine transaminase (ALT); (**L**) aspartate aminotransferase (AST); (**M**) triglycerides (TG); (**N**) cholesterol (TC); (**O**) low-density lipoprotein (LDL); (**P**) high-density lipoprotein (HDL) were tested. All results are presented as means ± standard error (SE). *n* = 6. * *p* < 0.05; ** *p* < 0.01; NS, no significance.

**Figure 3:**
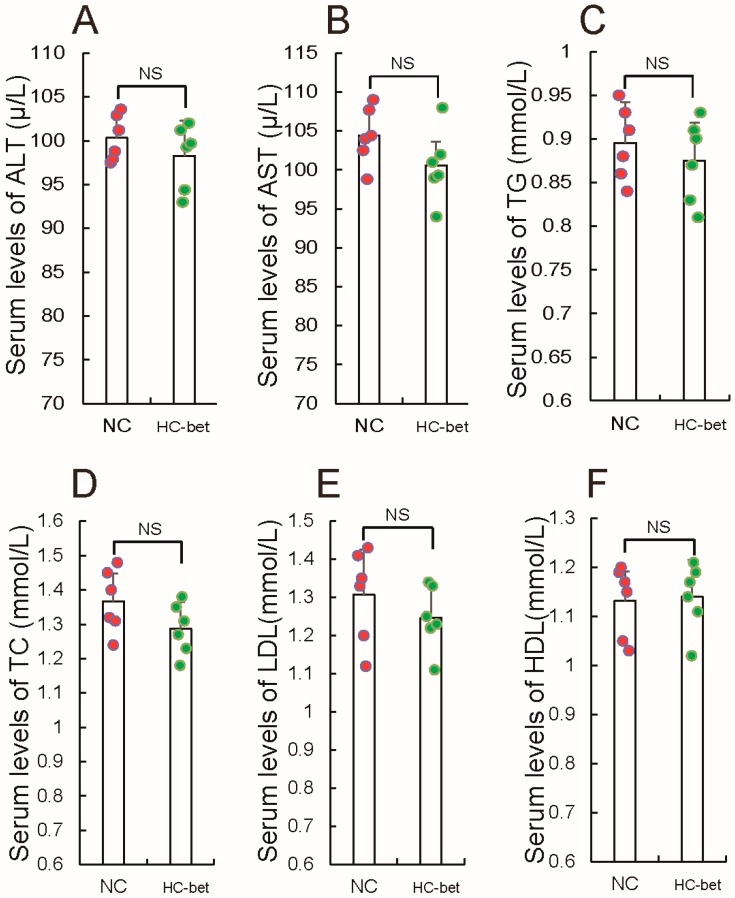
The effect of betaine supplementation on normal chow (NCW)-fed mice. NCW-fed mice were treated with 1% betaine in water (HC-bet) or without 1% betaine in water (NC), then serum levels of (**A**) alanine transaminase (ALT); (**B**) aspartate aminotransferase (AST); (**C**) triglycerides (TG); (**D**) cholesterol (TC); (**E**) low-density lipoprotein (LDL); (**F**) high-density lipoprotein (HDL) were tested. All results are presented as means ± standard error (SE). *n* = 6. * *p* < 0.05; ** *p* < 0.01; NS, no significance; NC, mice fed with normal chow; HC-bet, mice fed with normal chow and betaine.

Obesity is a major driver of some metabolic diseases, but there are no effective drugs to treat obesity. Recently, Ejaz et al. [32] reported that betaine could decrease inguinal WAT of mice fed with HFD, prompting the hypothesis that betaine supplementation can be considered for treatment of obesity. First, evaluation of the effect of betaine supplementation on adipocytes development, after mice fed with HFD or NCW were treated with or without 1% betaine in water was performed. This dose of betaine was intentionally chosen so as to align with previous studies that showed improved glucose homeostasis and prevention of fatty liver induced by a high-fat diet [32,45]. Figure 2A,B, in agreement with a previous report [32], shows betaine supplementation had little influence on the weight of NCW-fed mice, but significantly reduced body weight gain of HFD-fed mice. Expectantly, both HFD- and NCW-fed mice exhibited decreased inguinal (Figure 2C,D), gonadal (Figure 2E,F), as well as total fat mass (Figure 2G,H), when HFD- and NCW-fed mice were fed with 1% betaine in water, respectively. However, in contrast to the fact that 1% betaine supplementation significantly reduced perirenal fat mass in HFD-fed mice, 1% betaine supplementation had little influence on perirenal fat mass in HFD-fed mice (Figure 2I,J). Notably, in line with the observed resistance to HFD, 1% betaine supplementation displayed significant changes in plasma lipid and lipoprotein levels in HFD-fed mice. These include a significant decrease in plasma alanine transaminase (ALT) (Figure 2K), aspartate aminotransferase (AST) (Figure 2L), triglycerides (TG) (Figure 2M), cholesterol (TC) (Figure 2N) and low-density lipoprotein (LDL) (Figure 2O), and in contrast, a slight increase in plasma high-density lipoprotein (HDL) (Figure 2P). Meanwhile, we found that 1% betaine supplementation had no significant effect on plasma ALT, AST, LDL, HDL, TG and TC in NCW-fed mice compared to the control (Figure 3), which is consisted consistent with previous results [46,47]. Taken together, these results suggest that betaine supplementation in mice could inhibit WAT formation in vivo.

**Figure 4:**
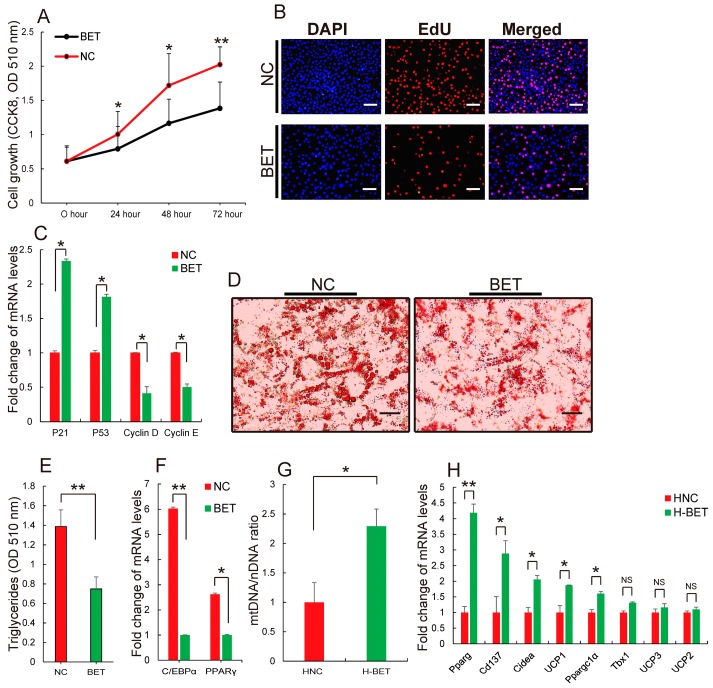
Betaine supplementation prevents the formation of new white adipose tissue (WAT), and guides original WAT burning in HFD-fed mice. Once 3T3-L1 cells were treated with 20 mM betaine, (**A**) cell proliferation was evaluated at 0 h, 24 h, 48 h and 72 h of proliferation by performing CCK8; (**B**) EdU proliferation assay was performed at 24 h to label proliferating cells; (**C**) The mRNA levels of genes related to cell proliferation were quantified. Additionally, 3T3-L1 cells were induced differentiation for 8 days; (**D**) cells were stained with oil red O; (**E**) triglycerides content was analyzed; (**F**) the mRNA levels of C/EBPα and PPARγ by qRT-PCR was measured and Scale bar, 10 μm. Meanwhile, after HFD-fed mice were fed with 1% betaine in water (H-BET) or without 1% betaine in water (HNC); (**G**) adipose tissue mitochondrial content was determined by the ratio of mtDNA/nDNA (*n* = 6); (**H**) qRT–PCR analysis of genes related to brown adipocyte (*n* = 6). All results are presented as means ± standard error (SE). Scale bar, 10 μm. * *p* < 0.05; ** *p* < 0.01; NS, no significance. BET, betaine; NC, negative control; H-BET, mice fed with high-fat diet and betaine; HNC, mice fed with high-fat diet.

Increased newly adipose tissue mass is ascribed to pre-adipocytes proliferation and differentiation, and the hypertrophy of mature adipocytes [48]. To explore whether betaine supplementation inhibits WAT formation through modulating pre-adipocytes proliferation and differentiation, 3T3-L1 white pre-adipocytes were treated with 20 mM betaine. First, CCK-8 and EdU assays were performed to evaluate the effect of betaine on the proliferation of 3T3-L1 white pre-adipocytes. As shown in Figure 4A, CCK8 analysis showed betaine treatment reduced the number of 3T3-L1 white pre-adipocytes, when compared to the untreated group. Furthermore, these results were further confirmed by an EdU assay. The ratio of EdU positive cells indicated the cell in DNA synthesis phage. Figure 4B shows the ratio of EdU positive cells was decreased remarkably after betaine treatment, suggesting that betaine might inhibit the proliferation of 3T3-L1 white pre-adipocytes. P21 is a cell cycle-arrest regulator, which is downstream of P53 [49,50]. Cyclin D/E are mammalian G1 cyclins that are both required and rate limiting for entry into S phase, and inhibition of cyclin D/E function can induce cell cycle arrest [51,52]. Agreeing with these observations, qRT-PCR analysis shows that betaine treatment caused an increase in the expression level of P21 and P53, while inhibiting cyclin D/E expression (Figure 4C). These results indicate that betaine might inhibit the proliferation of 3T3-L1 white pre-adipocytes by regulating cell cycle regulators. Subsequently, investigation of the effect of betaine on the differentiation of 3T3-L1 white pre-adipocytes were performed. The results showed that betaine treatment significantly reduced the number of oil red O+ cells (Figure 4D), triglyceride accumulation (Figure 4E), when compared to the control. C/EBPa and PPARγ, are two key transcription factors, which can mediate adipocyte differentiation and hypertrophy by regulating adipogenic gene expression [53,54]. Wu et al. [55] reported that mice deficient in C/EBPα have defective development of adipose tissue, for instance. Jones et al. [56] suggested that deletion of PPARγ in adipose tissues of mice protects against high fat diet-induced obesity lipid accumulation. Expectantly, qRT-PCR analysis suggested that C/EBPa and PPARγ were remarkably suppressed during adipocytes differentiation after betaine treatment (Figure 4F), suggesting that betaine could inhibit the differentiation of 3T3-L1 white pre-adipocytes, which is consistent with betaine being used as a dietary supplement in pig nutrition to reduce fat deposition [57]. Therefore, the above results confirm that inhibiting the proliferation and differentiation of pre-adipocytes could be considered as a reason that betaine supplementation decreases WAT formation.

Mature WAT contains most of the energy stored in the form of triglycerides (TGs), however, the potential mechanisms of how betaine consume mature WAT induced by HFD is still unclear. Recently, Lee et al. [58] reported that betaine treatment leads to an upregulation of mitochondrial respiration and cytochrome c oxidase activity in H2.35 cells. Surprisingly, estimated mitochondrial content by measuring the mtDNA/nDNA ratio, found that 1% betaine supplementation significantly increased the relative content of mitochondria in WAT of mice fed with HFD (Figure 4G). Moreover, in accordance with a previous report [32], the transcript levels of brown adipocyte markers were increased in WAT (Figure 4H), after mice fed with HFD were treated with 1% betaine in water. Mitochondria provide the majority of cellular energy in the form of ATP through oxidative phosphorylation (OXPHOS) [59]. Previously, numerous studies have revealed that WAT has fewer mitochondria [14,60]. Conversely, brown adipose tissue (BAT) contains dense mitochondria. Particularly, in response to an energy imbalance, BAT robustly enhances the whole animal energy expenditure in the form of heat [60,61,62]. Loss of BAT function is linked to obesity and metabolic diseases [63,64]. These data in this study suggest that betaine supplementation might consume WAT induced by HFD by stimulating mitochondrial biogenesis in WAT and promoting browning of WAT.

Taken together, the above findings indicate that preventing the formation of new WAT and guiding the original WAT burning, betaine supplementation inhibited WAT production in HFD-induced obese mice.

### 3.3. Betaine Supplementation Decreased Intramyocellular Lipid Accumulation in HFD-Induced Obese Mice

**Figure 5:**
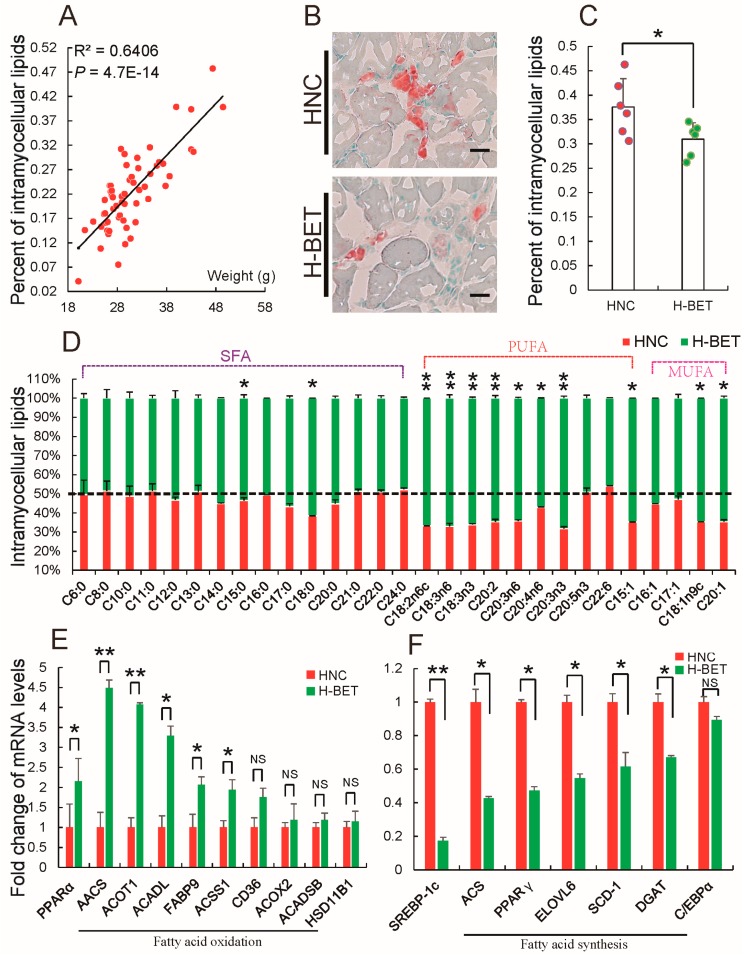
Betaine supplementation decreases intramyocellular lipid accumulation in high-fat diet (HFD)-induced obese mice. (**A**) Correlation between intramuscular fat contents and body weight, each point represents one mouse (*n* = 58). Once mice fed with a HFD were treated with or without 1% betaine in water; (**B**) the authors stained lipid droplets in muscle tissues with oil red O (Scale bar, 10 μm), and analyzed (**C**) intramuscular fat contents; (**D**) composition of intramuscular fatty acid (SFA, saturated fatty acid; PUFA, polyunsaturated fatty acids; MUFA, monounsaturated fatty acid), and the mRNA levels of genes associated with (**E**) fatty acid synthesis; (**F**) fatty acid oxidation in muscle tissues, these results are presented as means ± standard error (SE). *n* = 6. * *p* < 0.05; ** *p* < 0.01; NS, no significance; H-BET, mice fed with high-fat diet and betaine; HNC, mice fed with high-fat diet.

Obesity leads to accumulation of ectopic fat such as intrahepatic lipids and intramyocellular lipids [19,65]. Several previous results showed that betaine reduces hepatic triglyceride content [45]. To further evaluate the effect of betaine on HFD-induced obesity, the authors investigated whether betaine supplementation prevents accumulation of intramyocellular lipids, which is induced by HFD. Figure 5A is consistent with previous findings; intramyocellular lipid content positively correlated with body weight. It was found that 1% betaine supplementation prevented intramyocellular lipid accumulation induced by HFD (Figure 5B,C). Fatty acid is a main substrate of lipid metabolism. To confirm whether betaine reduced intramyocellular lipid accumulation induced by HFD is associated with fatty acid, comparison of fatty acid composition in muscle from HFD-induced mice treated with and without 1% betaine in water was performed. Figure 5D shows 1% betaine supplementation just increased a part of saturated fatty acids (SFA; C15:0 and C18:0), monounsaturated fatty acids (MUFA; C18:1n9c and C20:1), which is consistent with a previous study that found betaine supplementation could slightly increase content of SFA and MUFA in muscles from an obese pig breed [45]. Previously, some studies have proposed that SFA length and the physical characteristics of the triacylglycerol structure of SFA may impact lipoprotein metabolism [66]. These findings are a reminder that betaine supplementation might mediate lipid metabolism by regulating fatty acid composition in muscle from HFD-induced mice. Interestingly, further analysis found that almost all polyunsaturated fatty acids (PUFA) in intramyocellular lipids were significantly increased in muscle tissues, when HFD-fed mice were fed with 1% betaine (Figure 5D). This finding strongly confirmed that betaine supplementation could alter fatty acid composition in muscle of HFD-induced obese mice to mediate adipocytes development, because previous studies have demonstrated that PUFA mediates adipocyte proliferation, differentiation and energy metabolism by regulating the expression levels of genes related to lipid metabolism [67,68,69]. Particularly, PUFA represses fatty acid synthesis by decreasing the expression of SREBP-1c, whereas it enhances fatty acid oxidation by activating PPARα [70,71]. The authors thus evaluated whether betaine supplementation could regulate the synthesis and oxidation of fatty acid in intramyocellular lipids of HFD-fed mice. A qRT-PCR analysis showed that 1% betaine supplementation up-regulated PPARα expression (Figure 5E) but down-regulated SREBP-1c expression (Figure 5F). Conformably, 1% betaine supplementation enhanced genes associated with fatty acid oxidation (Figure 5E), whereas inhibited genes were involved with fatty acid synthesis (Figure 5F), suggesting that, by increasing PUFA to promote fatty acid oxidation but inhibit synthesis, betaine supplementation might reduce intramyocellular lipid accumulation in HFD-induced obese mice. Taken together, the above results indicate that, by mediating fatty acid metabolism, betaine supplementation reduced intramyocellular lipid accumulation in HFD-induced obese mice.

### 3.4. Betaine Supplementation Relieved Inflammation and Improved Insulin Resistance in HFD-Induced Obese Mice

**Figure 6:**
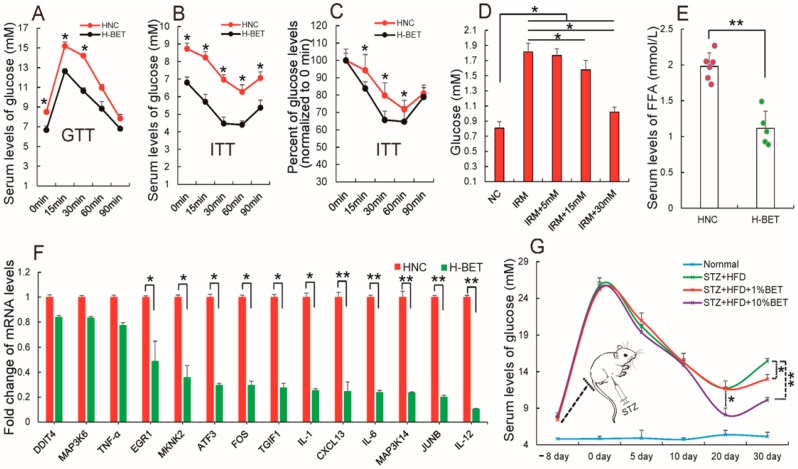
Betaine (BET) supplementation improves insulin resistance. Once mice fed with high-fat diet (HFD) were treated with or without 1% or 10% betaine in water, (**A**) glucose-tolerance tests (GTT) and (**B**) insulin-tolerance tests (ITT) in HFD feeding mice treated with or without betaine were performed; (**C**) Changes of glycemia level on the ITT were indicated as percentage; (**D**) The glucose level of medium cultured insulin-resistant 3T3-L1 cells; (**E**) The serum free fatty acids (FFAs) levels and (**F**) the mRNA levels of some inflammatory stress-related genes in adipose tissues were analyzed; (**G**) Additionally, the serum level of glucose in normal chow (NCW)-fed mice (normal), STZ-injected mice fed HFD which were treated without (streptozotocin (STA) + HFD) and with different does of betaine (STA + HFD + BET) were measured. All results in vivo are presented as means ± standard error (SE). *n* = 6. * *p* < 0.05; ** *p* < 0.01; H-BET, mice fed with HFD and betaine; HNC, mice fed with HFD.

Insulin resistance is a common characteristic associated with obesity [72]. To confirm that betaine supplementation limited HFD-induced obesity, a GTT and ITT to evaluate insulin sensitivity in HFD-induced obese mice fed with and without betaine in water. Figure 6A,B demonstrates that 1% dietary betaine supplementation reduced values in insulin-tolerance tests (ITTs), whereas it improved glucose metabolism in glucose-tolerance tests (GTTs) in HFD-induced mice, as compared to the control group. Consistently, the change of glycemia levels on the ITTs were further evaluated as percentage, and found that the percent of decrease glycemia is greater in experimental group than control group, indicating that betaine supplementation may improve insulin sensitivity in HFD-induce obese mice (Figure 6C). Subsequently, to further confirm these results in vivo, an insulin-resistant adipocyte model according to the method described previously by Xu et al. [73] was established. Figure 6D shows betaine treatment (20 mM) increased glucose uptake by measuring the glucose levels of medium cultured insulin-resistant 3T3-L1 cells, suggesting that betaine treatment improved insulin resistance in an insulin-resistant adipocyte model. Previously, some studies revealed that obesity-induced inflammation, especially in obese adipose tissue, is an important cause of obesity-induced insulin resistance [42,74,75,76]. The rise in FFAs levels lead to proinflammatory gene expression and decreases sensitivity to insulin [44,77,78]. Consistent with betaine supplementation improving insulin resistance, it also was observed that 1% betaine supplementation decreased the level of serum free fatty acids (FFAs) in HFD-fed mice (Figure 6E), a result consistent with previous reports [79]. Meanwhile, 1% betaine supplementation caused a significant decrease in the expression levels of some inflammatory stress-related factors in adipose tissues of HFD-fed mice (Figure 6F), which has been reported in human adipocytes where betaine reduced hypoxia-induced expression of inflammatory adipokines [80]. These results demonstrate that betaine supplementation improves insulin resistance in HFD-induced obese mice.

Increased insulin resistance causes hyperglycemia, which is a major metabolic abnormality in a great majority of patients with type 2 diabetes. To examine the possibility of dietary betaine supplementation in the treatment of hyperglycemia, mice were injected with streptozotocin (STZ), which induces hyperglycemia and even diabetes in vivo. Figure 6G shows STZ successfully induced hyperglycemia. The STZ diabetic mice were then treated with betaine in water for 30 days and the blood glucose levels were found to decrease. Taken together, these data provide evidence that dietary betaine supplementation might improve obesity and non-obesity induced insulin resistance.

## 4. Conclusions

To summarize, the present study showed that betaine treatment not only significantly inhibited the proliferation and differentiation of 3T3-L1 adipocytes in vitro, but also remarkably suppressed accumulation of white adipose tissues such as inguinal, gonadal and perirenal fat in vivo. Following betaine supplementation, HFD-fed mice displayed a significant decrease in plasma lipid and lipoprotein levels, such as TG and LDL. Nevertheless, no significant differences were observed in HDL. Additionally, the authors found that positive regulation of the relative number of mitochondria, browning of WAT and FAO, and reduced expression of some genes promoted lipid synthesis in WAT of HFD-fed mice. This mechanism was mediated by betaine in HFD-fed mice. More importantly, dietary betaine supplementation was shown to reduce intramyocellular lipid accumulation and improve obesity induced insulin resistance. Therefore, dietary betaine supplementation can be considered as a possible therapeutic approach for obesity and hyperglycemia.

## Supplementary Materials

The following are available online at http://www.mdpi.com/2072-6643/10/2/131/s1, Table S1, The primer sequences used for qPCR.
