# Supplementary material for: Betaine Supplementation Enhances Lipid Metabolism and Improves Insulin Resistance in Mice Fed a High-Fat Diet
Source: Nutrients. 2018 Jan 26;10(2):131. doi: 10.3390/nu10020131 (PMC5852707; doi:10.3390/nu10020131)
Supplement: Supplementary file 1 [file nutrients-10-00131-s001.pdf]

Supplementary Table S1. The primer sequences used for qPCR.

Gene	Primer sequence (5`→3`)
PPARγ	F: CTCCAAGAATACCAAAGTGCGA
	R: GCCTGATGCTTTATCCCCACA
SREBP-1c	F: GCAGCCACCATCTAGCCTG
	R: CAGCAGTGAGTCTGCCTTGAT
C/EBPα	F: CAAGAACAGCAACGAGTACCG
	R: GTCACTGGTCAACTCCAGCAC
ELOVL6	F: AAGCACGCTCTATCTCCTGTT
	R: CTGCGTTGTATGATCCCATGAA
ACS	F: CGCACCCTTCCAACCAACA
	R: CGCTATTTCCACTGACTGCAT
SCD-1	F: TTCTTGCGATACACTCTGGTGC
	R: CGGGATTGAATGTTCTTGTCGT
DGAT	F: CTGATCCTGAGTAATGCAAGGTT
	R: TGGATGCAATAATCACGCATGG
P53	F: AAACGCTTCGAGATGTTCCG
	R: GTAGACTGGCCCTTCTTGGT
P21	F: GATGGCTTCGACACCATTCC
	R: AGACGACACAGGTGAGGAAG
Cyclin D	F: TAGGCCCTCAGCCTCACTC
	R: CCACCCCTGGGATAAAGCAC
Cyclin E	F: CAGAGCAGCGAGCAGGAGC
	R: GCAGCTGCTTCCACACCACT
Ucp1	F: CTTTGCCTCACTCAGGATTGG
	R: ACTGCCACACCTCCAGTCATT
Ucp2	F: CAGGTCACTGTGCCCTTACCAT
	R: CACTACGTTCCAGGATCCCAAG
Ucp3	F: GCCTTCTCTCTCGGAGGTTT
	R: GCAGATGGAAGACTGAAGGC
Cidea	F: TATGTCCCAGTCTGCAAGCA
	R: TAACACGGCCTTGAAGCTTG
Tbx1	F: TGGGACGAGTTCAATCAGCT
	R: ATACCGGTAGCGCTTGTCAT
Cd137	F: TGAAATTCAGGTGCTGCAGG
	R: TGCAGAAAGTACCAGGCTGA
Pparg	F: AGGGCGATCTTGACAGGAAA
	R: CGAAACTGGCACCCTTGAAA
Ppargc1α	F: AGCCTCTTTGCCCAGATCTT
	R: TCTGTGAGAACCGCTAGCAA
PPARα	F: ACCTTGTGTATGGCCGAGAA
	R: AAGGAGGACAGCATCGTGAA

AACS	F: ACGGCAACATGACAAGCAGT
	R: ACAAGGTCCCACAACACGTT
ACOT1	F: TGGTTTGGAGGTTGGGGAAA
	R: AAACTCCATTCCCAGCCCTT
ACADL	F: ACATACAGACGGTGCAGCAT
	R: TCCGTGGAGTTGCACACATT
FABP9	F: TGCACGATGATTGAGCCCTT
	R: TGTTCCTGCATGCACTTCCT
ACSS1	F: TTGGTGGCAAACGGAAACTG
	R: ACCCTCCAAAACATTGCCCT
CD36	F: TGCTCAGGATGTCAATGGCT
	R: TAGAACAGCTTGCTTGCCCA
ACOX2	F: AAACACTGAGCTGCGGAGAA
	R: AATGCGTTCAGGACCGTCTT
ACADSB	F: TAAACAGCCTGCCCTCCTTT
	R: TCAGCACTGGAAGCCTGAAA
HSD11B1	F: ACCAGAAATGCTCCAGGGAA
	R: TGCCAGCAATGTAGTGAGCA
DDIT4	F: ACCGGCTTCAGAGTCATCAA
	R: TCAGGTGGCTATCGTCAGTT
MAP3K6	F: ACTCCTTCTACAACGCGGAT
	R: TGCCAACACAATCCGAGTTC
TNFα	F: TGGAGCCTCGAATGTCCATT
	R: ACCCCGGCCTTCCAAATAAA
EGR1	F: ATCCCAGCTCATCAAACCCA
	R: CGACTGAAGTTACGCATGCA
MKNK2	F: TTCGAGTTGGCCTTCTCCTT
	R: ACATCTTCGAACCTGCCTGA
ATF3	F: TTGTCCAATGGCCAGGGTAT
	R: TGCTTTTGAGGGGCACTTTC
FOS	F: AGCGAGCAACTGAGAAGACT
	R: GCGTTGAAACCCGAGAACAT
TGIF1	F: TGCGAGACTGGCTGTATGAA
	R: GCGGGAAATCGTGAACTGAT
IL-1	F: TTCCTGAAGCAGCTGAGTGT
	R: CAGCAGCTGGTTATTGTGCA
CXCL13	F: ATCATGAGGTGGTGCAAAGC
	R: ATCATCAGGGTCACAGTGCA
IL-6	F: ACAAAGCCAGAGTCCTTCAGA
	R: ATGGTCTTGGTCCTTAGCCA
MAP3K14	F: TCTCTCAATGGCGAACACCT
	R: TTCTCCAGCTGACCATGCTT
JUNB	F: AGAAAATCTCCGGAAGCCCA

	R: TCCATTTTCGTGCACATCCG
IL-12	F: TTGAAAGGCTGGGTATCGGT<br>R: AGCCAGCTCCTCATGAATGT
β-actin	F: TGGAATCCTGTGGCATC CATGAAAC<br>R: TAAAACGCAGCTCAG TAACAGTCCG

F: forward, R: reverse. *β-actin* was used as endogenous control genes for mRNA.
